# Membrane Dynamics and Organization of the Phagocyte NADPH Oxidase in PLB-985 Cells

**DOI:** 10.3389/fcell.2020.608600

**Published:** 2020-11-12

**Authors:** Jérémy Joly, Elodie Hudik, Sandrine Lecart, Dirk Roos, Paul Verkuijlen, Dominik Wrona, Ulrich Siler, Janine Reichenbach, Oliver Nüsse, Sophie Dupré-Crochet

**Affiliations:** ^1^Université Paris-Saclay, CNRS U8000, Institut de Chimie Physique, Orsay, France; ^2^Light Microscopy Core Facility, Imagerie-Gif, Institut de Biologie Intégrative de la Cellule (I2BC), CEA, CNRS, Université Paris-Saclay, Gif-sur-Yvette, France; ^3^Sanquin Research and Landsteiner Laboratory, Amsterdam University Medical Center, University of Amsterdam, Amsterdam, Netherlands; ^4^Division of Gene and Cell Therapy, Institute for Regenerative Medecine, University of Zurich, Zurich, Switzerland

**Keywords:** phagocytosis, NOX2, super-resolution imaging, dSTORM, nanoclusters

## Abstract

Neutrophils are the first cells recruited at the site of infections, where they phagocytose the pathogens. Inside the phagosome, pathogens are killed by proteolytic enzymes that are delivered to the phagosome following granule fusion, and by reactive oxygen species (ROS) produced by the NADPH oxidase. The NADPH oxidase complex comprises membrane proteins (NOX2 and p22^phox^), cytoplasmic subunits (p67^phox^, p47^phox^, and p40^phox^) and the small GTPase Rac. These subunits assemble at the phagosomal membrane upon phagocytosis. In resting neutrophils the catalytic subunit NOX2 is mainly present at the plasma membrane and in the specific granules. We show here that NOX2 is also present in early and recycling endosomes in human neutrophils and in the neutrophil-like cell line PLB-985 expressing GFP-NOX2. In the latter cells, an increase in NOX2 at the phagosomal membrane was detected by live-imaging after phagosome closure, probably due to fusion of endosomes with the phagosome. Using super-resolution microscopy in PLB-985 WT cells, we observed that NOX2 forms discrete clusters in the plasma membrane. The number of clusters increased during frustrated phagocytosis. In PLB-985*NCF1*ΔGT cells that lack p47^phox^ and do not assemble a functional NADPH oxidase, the number of clusters remained stable during phagocytosis. Our data suggest a role for p47^phox^ and possibly ROS production in NOX2 recruitment at the phagosome.

## Introduction

The phagocytic NADPH oxidase produces reactive oxygen species (ROS) that are crucial for killing pathogens during phagocytosis. NADPH oxidase is a multi-subunit enzyme comprising several membrane and cytosolic components, the flavocytochrome *b*_558_ (NOX2) and p22^phox^ in membranes, and the cytosolic subunits (p47^phox^, p67^phox^, and p40^phox^) and the small GTPase Rac. Upon phagocytosis, the cytosolic subunits and Rac assemble with the membrane subunits. This translocation allows electron flow from NADPH in the cytosol to oxygen in the phagosome, through the catalytic subunit NOX2, leading to superoxide anion production, which is the precursor of other types of ROS (Nunes et al., 2013; Valenta et al., 2020). A lack of a functional NADPH oxidase such as in chronic granulomatous disease results in life-threatening infections with bacteria and fungi (O’Neill et al., 2015). Thus, ROS production is necessary for the immune response against many pathogens. NOX2 forms a heterodimer with p22^phox^ in the endoplasmic reticulum. The heterodimer formation is necessary for its trafficking in the Golgi, where NOX2 is further glycosylated (DeLeo et al., 2000). In neutrophils, the heterodimer has been localized in the plasma membrane, in specific and gelatinase granules, and in secretory vesicles (Borregaard et al., 1983; Lominadze et al., 2005). In macrophages, the heterodimer is present in the plasma membrane and in Rab5 and Rab11-positive endosomes (Casbon et al., 2009). Since the early and recycling endosomes fuse with the phagosome during phagocytosis (Niedergang et al., 2003; Pauwels et al., 2017), they may add new heterodimers within the phagosomal membrane.

In order to explore whether the endosomal pathway contributes to the enrichment in phagosomal NOX2 in neutrophils, as in macrophages, we used the neutrophil-like PLB-985 cells that express neither specific and gelatinase granules nor secretory vesicles (Pivot-Pajot et al., 2010; Rincón et al., 2018). First, using live imaging and the X^0^-CGD PLB-985 cell line expressing GFP-NOX2 (van Manen et al., 2008), we observed an increase in phagosomal NOX2 after phagosomal closure. Immunofluorescence studies indicated that PLB-985 cells, as well as primary human neutrophils, contained early and recycling endosomes positive for NOX2. During phagocytosis, these endosomes were in close contact with the phagosome suggesting that they could contribute to the phagosomal gain in NOX2. Moreover, using direct stochastic optical resolution microscopy (dSTORM) in a total internal reflection fluorescence microscopy (TIRFM) configuration, we observed that NOX2 formed discrete clusters during frustrated phagocytosis. The number of clusters increased during phagocytosis in PLB-985 WT cells but not in PLB-985 NCF1ΔGT cells that lack a functional oxidase due to the absence of p47^phox^ (Wrona et al., 2017). These data suggest that the presence of p47^phox^ and/or NADPH oxidase activity triggers a positive feedback with the recruitment of NOX2 positive vesicles.

## Materials and Methods

### Cell Culture

Several PLB-985 cell lines were used in this study. The human myeloid leukemia cell lines PLB-985 WT was a generous gift from Dr. Marie-José Stasia (Faculty of Medecine, Université de Grenoble Alpes, France). The X^0^-CGD PLB-985 GFP-NOX2 cell line corresponds to PLB-985 WT cells deleted for endogenous NOX2 (Zhen et al., 1993) but expressing GFP-NOX2 (van Manen et al., 2008). PLB-985 NCF1ΔGT cells lack a functional oxidase due to deletion of a dinucleotide in the *NCF1* gene, encoding p47^phox^, by CRISPR/Cas9 manipulation (Wrona et al., 2017). PLB-985 cells were cultured and differentiated for 5 or 6 days with 1.25% DMSO as previously described (Song et al., 2020). IFN-γ (2000 U/ml, 11343536, Immunotools) was added to the culture 24 h before the experiments.

### Neutrophil Preparation

Human blood samples were taken with the understanding and written consent of each volunteer by the “Etablissement Français du Sang, Cabanel, Paris” (the French blood transfusion service and National Blood Bank: https://www.ints.fr/). An agreement (N°11/Necker/103) allowing us to use blood samples from the volunteers for research purposes was signed between this organization and our laboratory. Neutrophils were isolated from healthy donor whole blood as previously described (El Benna et al., 1997). For all the experiments, the cells were resuspended in Hank’s Balanced Salt Solution (H8624, Sigma-Aldrich).

### Opsonization of Zymosan and Phagocytosis

Zymosan (Z2849, Invitrogen^TM^) and Texas-Red-zymosan (Z2843, Invitrogen^TM^) from *S. cerevisiae* were opsonized with human serum (diluted 50%, H4522, Sigma-Aldrich) as described previously (Tlili et al., 2011).

For live-imaging, zymosan particles were added directly to 5 × 10^5^ cells (5 particles per cell). For short-term live-imaging, a Z-stack with an increment of 0.5 μm was taken, from the start of phagocytosis, every 30 s. For longer term live-imaging (>3 min), the contact between zymosan and cells was obtained by centrifugation at 13°C at 400 *g* during 3 min. The end of the centrifugation was defined as Time Zero for the phagocytosis. A Z-stack with an increment of 1 μm was made every 5 min.

### Frustrated Phagocytosis

For super-resolution microscopy, coverslips were washed for 30 min with 0.1% (w/v) Decon 90 and 100 mM sodium hydroxide under sonication. The coverslips were then kept in 2 N sodium hydroxide for 2 h before washing with pure water followed by 70% ethanol. Finally, they were soaked for 1 h in absolute ethanol followed by an acetone wash and then dried in an oven for 30 min at 70°C. For frustrated phagocytosis, coverslips were coated with BSA (10 mg/ml, B1529, Sigma-Aldrich) overnight. After a washing step, a rabbit anti-BSA antibody was added (1:500, B1520, Sigma-Aldrich) at room temperature for 1 h. Cell adhesion on poly-lysine (P4707, Sigma-Aldrich) coated coverslip was carried out as described previously (Mularski et al., 2018).

### Immunofluorescence

The immunofluorescence experiments were performed as described previously (Tlili et al., 2012) except that, after paraformaldehyde fixation, the coverslips were incubated 5 min twice with 10% (w/v) glycine in PBS. The cells were immunostained with a rabbit anti-EEA1 antibody (1:100, PA5-17228, Invitrogen) or a rabbit anti-Rab11 (1-5μg, 71-5300, Thermofisher) and a mouse anti-NOX2 antibody (1:1000, Abcam, ab80897), followed by Alexa-488 goat anti-mouse antibody (1:1000, A11029, Life Technologies) or Dylight 405 goat anti-rabbit (1:200, 35550, Thermofisher).

The same protocol was used for dSTORM experiments, the only difference concerned the secondary antibody. After the mouse anti-NOX2 antibody, a mix with Alexa-647 F(ab′)_2_ goat anti-mouse Immunoglobulin G (IgG; 1:3000, A21237, Thermo Fisher Scientific) and a blocking antibody against free rabbit anti-BSA antibody (1:1000, ab6831, Abcam) were used during 1 h at room temperature.

### Microscopy

Imaging was carried out with a Spinning-Disk Confocal System (Yokogawa CSU-X1-A1, Yokogawa Electric, Yokogawa, Japan), mounted on a Nikon Eclipse Ti E inverted microscope, equipped with a 100x APO 1.4 oil immersion objective and an EM-CCD eVolve 512 camera (Photometrics), driven by MetaMorph 7 software (Molecular Devices). GFP-NOX2 protein was excited at 491 nm (Cobolt Calypso, 100 mW) with an exposure time of 200 ms. Fluorescence was detected with a double-band beam splitter (491–561 nm) and a 525/45-nm emission filter. For immunofluorescence, 561 nm (Coherent, 100 mW) and 405 nm (Vortran, 100 mW) lasers were also used with a quad-band beam splitter and a quad band emission filter (440/40 nm, 521/20 nm, 607/34 nm, 700/45 nm, Semrock).

DSTORM super-resolution microscopy is based on stochastic blinking of individual fluorophores. Each detection sequence detects a small number of fluorophores in the microscope field. A large number of images (20000) was recorded in order to detect each individual blinking fluorophore and to reconstruct images with high spatial precision. To achieve the blinking process for dSTORM, the coverslips (thickness 0.17 mm) were incubated in a specific imaging buffer composed of 0.63 mg/ml glucose oxidase (G2133, Sigma-Aldrich) and its substrate (0.1 g/ml glucose), 40 μg/ml catalase (C100, Sigma-Aldrich) and 110 mM mercaptoethylamine. Depletion in oxygen by the glucose oxidase, and the thiols stabilize the fluorophores in a dark state thereby reducing the number of fluorophores that are blinking at the same time (Endesfelder and Heilemann, 2015). Imaging was carried out with a Nikon Eclipse Ti E inverted microscope, equipped with a motorized *x*,*y*,*z* perfect focus system and a 100x APO TIRF SR (N.A. 1.49) oil immersion objective and an Andor iXon Ultra 897 EM-CCD camera driven by NIS-Elements Advanced Research software (Nikon). A 647 nm laser (MPB Communication, 300 mW) and a 405 nm diode (Cube, Coherent, 100 mW) were used. The fluorescence was detected with a quad band emission filter (450/60-525/50-605/50-730/120, Chroma). 20,000 images, each with an exposure of 16 ms were taken per acquisition.

### Image Processing and Analysis

Image J software was used to analyze immunofluorescence images, to quantify fluorescence in real-time microscopy and to determine the cell surface after frustrated phagocytosis. A line over plasma and phagosomal membrane was drawn and the ratio between fluorescence at the phagosomal membrane (minus background) and fluorescence at the plasma membrane (minus background) was calculated for each time point. Co-localization was analyzed using the JACoP ImageJ plugin (Bolte and Cordelières, 2006). A negative control was made by recalculating the Pearson coefficient using the NOX2 image rotated by 180° (Dunn et al., 2011).

Direct stochastic optical resolution microscopy analysis was performed using thunderSTORM software (Ovesný et al., 2014). First, images were filtered using the “difference of averaging” filter. Blinking events were detected using the local maximum method and then sub-pixel localization was estimated using a point spread function model (PSF integrated Gaussian). The detection process thus provided a list of every fluorophore with an estimation of the number of emitted photons and their precise localization. Artifactual detections were avoided by removing all molecules whose localization lacked precision, having a variability greater than 20 nm. We estimated the maximal radius of a sphere containing the NOX2 plus antibody-complex to be 20 nm since the radius of NOX2 can be estimated as 3 nm (Erickson, 2009), the length of the primary antibody is 10-15 nm and the F(ab′)_2_ fragment of the IgG is 5 nm (Hainfeld and Powell, 2000).

Using SR-Tesseler software (Levet et al., 2015) the molecule list was then analyzed with the density based spatial clustering of applications with noise algorithms (DBSCAN, Ester et al., 1996) used to detect cluster organization. The algorithms gather points that are close together taking into account a minimum number of fluorophores within a minimum distance. We considered as the minimal distance the estimated diameter of NOX2 plus the antibody complex (40 nm). The minimum number of fluorophores in clusters was 15 in order to get at least two molecules of NOX2 in one cluster. Indeed one Alexa-647 F(ab′)_2_ goat anti-mouse IgG can carry up to 7 Alexa-647 molecules, and we assumed that 2 Alexa 647 F(ab′)_2_ fragment can bind one primary anti-NOX2 antibody. The algorithm thus gave a list of clusters indicating their position, size and the number of fluorophores in each cluster, making it possible to compare different experimental conditions.

### Data Analysis

Graphpad prism8 software (GraphPad Software, United States) was used for statistical analyses. Since the values were not normally distributed, Mann–Whitney tests were performed to compare 2 groups, and Kruskal Wallis tests were used to compare multiple groups. For the live-imaging experiments, ratio-paired *t*-tests were used to compare fluorescence ratios at the different times. Differences were considered significant when *p* < 0.05. Statistical test results are indicated in the figure legends.

## Results

### Phagosomal NOX2 Level Increases After Phagosome Closure

**FIGURE 1:**
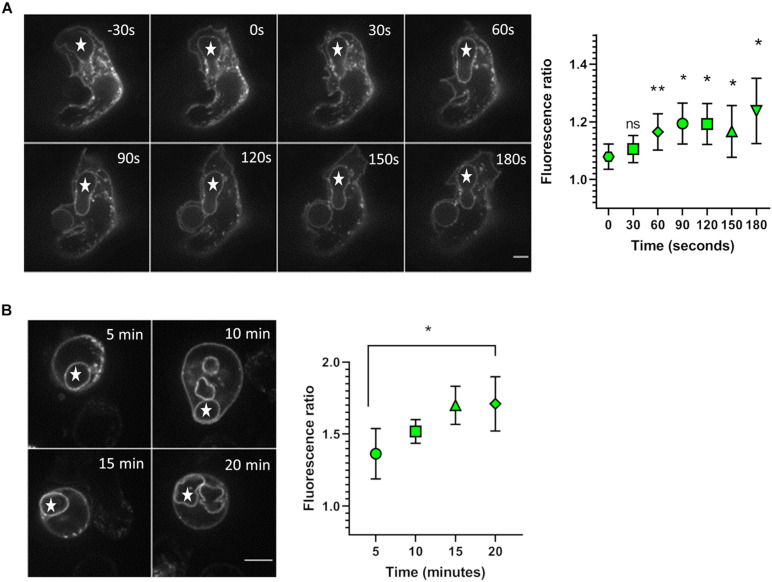
Accumulation of NOX2 in the phagosomal membrane. **(A)** X^0^-CGD PLB-985 GFP-NOX2 cells were incubated with opsonized zymosan and the phagocytosis was observed by spinning disk confocal video-microscopy. Left: representative images of zymosan (*) phagocytosis at the indicated times. Time 0 represents the closure of the phagosome. The images shown are single planes from a Z-stack. Scale bar = 5 μm. Right: kinetics of NOX2 accumulation at the phagosome. The fluorescence ratio represents the ratio of NOX2 fluorescence intensity at the phagosome to that at the plasma membrane. Data are means ± SEM. Nineteen videos were analyzed from three independent experiments, *p < 0.05, **p < 0.01 (ratio paired *t*-test comparing value at the indicated time with that at time 0). **(B)** X^0^-CGD PLB-985 GFP-NOX2 cells were incubated with opsonized zymosan and immediately centrifuged at 13°C. The end of centrifugation corresponds to time 0. Phagocytosis was then observed, at 37°C, using spinning disk confocal microscopy, a Z-stack was taken every 5 min. Left: representative images of zymosan (*) phagocytosis. The images shown are single planes from a Z-stack. Scale bar = 5 μm. Right: kinetics of NOX2 accumulation at the phagosome between 5 and 20 min. Data are means ± SEM. Sixteen cells were analyzed from four independent experiments, *p < 0.05 (ratio paired *t*-test).

We first investigated whether the level of NOX2 in the phagosomal membrane increases during phagocytosis in PLB-985 cells. The NOX2 increase could be due to lateral diffusion of NOX2 protein in the plasma membrane, which might then accumulate in the phagosomal cup, or to fusion of NOX2-containing organelles with the phagosomes. X^0^-CGD PLB-985 GFP-NOX2 were used to follow GFP-NOX2 accumulation in the phagosomal membrane. X^0^-CGD PLB-985 cells do not express NOX2, thus we checked that expression of GFP-NOX2 in these cells was able to restore ROS production upon phagocytosis of serum opsonized zymosan in our luminometry assay using L-012 although the production was a bit reduced as compared to PLB-WT cells (unpublished data). GFP fluorescence was located at the plasma membrane and also in discrete dots inside the cells (Figure 1A). The phagocytosis of opsonized zymosan was followed using 4D live imaging during the first 180 s after the phagosome closure (Figure 1A). At the onset of phagocytosis, the fluorescence ratio of GFP-NOX2 between the phagosomal membrane and the plasma membrane was about 1, indicating that the phagosomal level of NOX2 did not increase before phagosome sealing. However, 1 min after phagosomal closure a gain in phagosomal membrane NOX2 was measured. To observe GFP-NOX2 over a longer period of time after phagosomal closure, we synchronized the phagocytosis by centrifugation and followed the phagosome maturation using 4D live imaging (Figure 1B). At 5 min after the centrifugation, the fluorescence ratio was 1.4 (±0.18), close to that obtained in the previous experiment 3 min after phagosome closure. This ratio further increased to 1.7 (±0.19) after 20 min of phagocytosis, indicating that the phagosomal membrane had gained new NOX2 molecules. Our data thus indicate that in PLB-985 cells part of GFP-NOX2 was recruited within the phagosomal membrane after phagosome sealing, suggesting that some organelles can deliver new NOX2 molecules to the phagosome.

### NOX2 Is Localized in Some EEA1 and Rab11-Positive Endosomes

**FIGURE 2:**
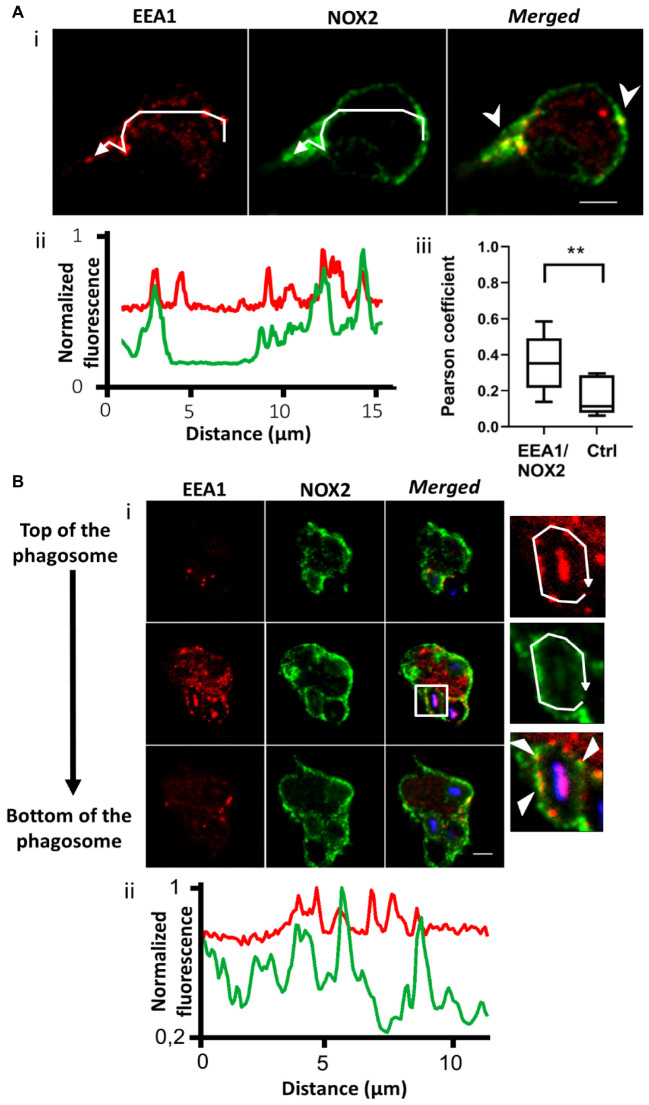
NOX2 is present in some EEA1-positive endosomes that localize close to the phagosome during phagocytosis. **(A)** EEA1 (red) and NOX2 (green) were detected by immunofluorescence in resting PLB-985 WT cells using spinning disk confocal microscopy. **(Ai)** Representative images of single planes from a Z-stack. Scale bar = 3 μm. Three independent experiments. **(Aii)** Normalized fluorescence of EEA1 and NOX2 along the white arrow shown in **Ai**. Overlapping peaks of fluorescence indicate localization of NOX2 and EEA1 in the same structures. **(Aiii)** Co-localization of EEA1 and NOX2 estimated using the Pearson coefficient (PC) calculated with the JACoP plug-in in Image J. PCs correlating EEA1 and NOX2 images (EEA1/NOX2) were controlled by comparison with the PCs correlating the EEA1 image and the image of NOX2 rotated by 180° (Control, Ctrl). Eight images from three independent experiments were analyzed. Each boxplot represents the inter-quartile range with the median, ** represents *p* < 0.01 (Mann Whitney test). **(B)** EEA1 (red) and NOX2 (green) were detected by immunofluorescence after 10 min of phagocytosis with opsonized Texas Red-zymosan. **(Bi)** Three planes from a series of Z-stack planes (0.5 μm). Some EEA1 endosomes were observed close to or at the phagosomes. Scale bar = 3 μm. Three independent experiments. **(Bii)** Normalized fluorescence of EEA1 and NOX2 along the white arrow showing some overlapping peaks of NOX2 and EEA1 fluorescence at the phagosome.

**FIGURE 3:**
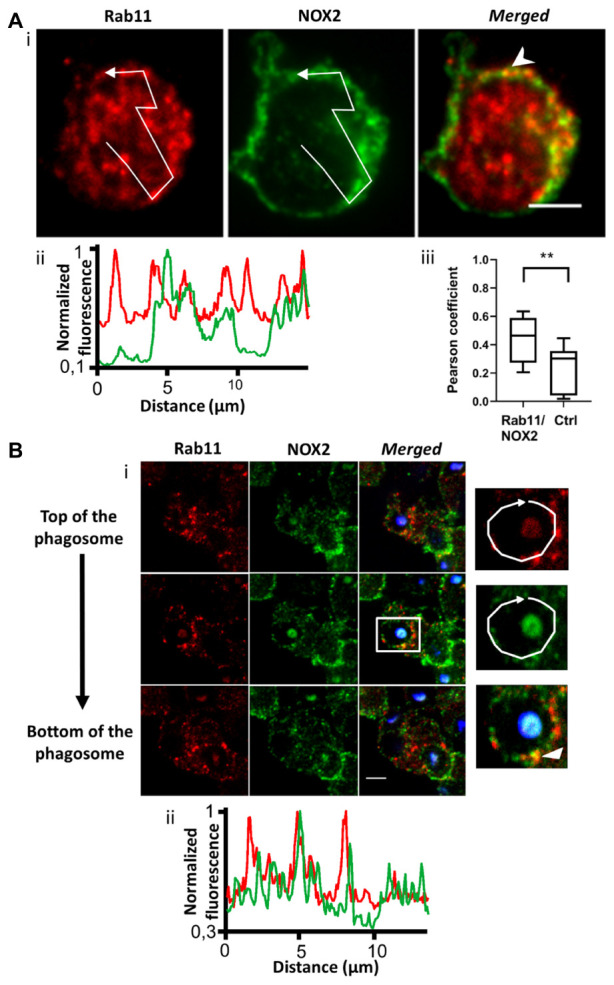
NOX2 is present in some Rab11-positive endosomes that are localized close to the phagosome during phagocytosis. **(A)** Rab11 (red) and NOX2 (green) were detected by immunofluorescence in resting PLB-985 WT cells using spinning disk confocal microscopy. **(Ai)** Representative images of single planes from a Z-stack. Scale bar = 3 μm. Three independent experiments. **(Aii)** Normalized fluorescence of Rab11 and NOX2 along the white arrow shown in **Ai**. Overlapping peaks of fluorescence indicate co-localization of NOX2 and Rab11 in the same structures. **(Aiii)** Co-localization between Rab11 and NOX2 was estimated using the Pearson coefficient (PC) calculated with the JACoP plug-in in Image J. PCs correlating Rab11 and NOX2 images (Rab11/NOX2) were controlled by comparison with the PCs correlating the Rab11 image and the image of NOX2 rotated by 180° (Control, Ctrl). Eight images were analyzed from three independent experiments. Each boxplot represents the inter-quartile range with the median, ** represents *p* < 0.01 (Mann Whitney test). **(B)** Rab11 (red) and NOX2 (green) were detected by immunofluorescence after 10 min of phagocytosis with opsonized Texas Red-zymosan. **(Bi)** Three planes from a Z-stack series (0.5 μm). Some Rab11 structures were observed close to or at the phagosomes. Scale bar = 3 μm. Three independent experiments. **(Bii)** Normalized fluorescence of Rab11 and NOX2 along the white arrow showing some overlapping peaks at the phagosome.

The endosomal compartment is a potential source for NOX2 delivery. Indeed, upon endotoxin stimulation, NADPH oxidase has been described to assemble at the early endosome (Lamb et al., 2012). We used immunofluorescence to detect NOX2 in early and recycling endosomes in resting PLB-985 cells and neutrophils. We observed NOX2 and early endosome antigen1 (EEA1) co-localization. EEA1 is a marker of early endosomes (Mu et al., 1995). NOX2 was present in some EEA1-positive endosomes in PLB-985 WT cells and neutrophils (Figures 2Ai,ii and Supplementary Figures 1Ai,ii). Using the JACoP plugin in Image J, we determined that (1) the Pearson coefficient was 0.35 (±0.05), indicating partial co-localization, and (2) that 27.3% of the EEA1-positive endosomes contained NOX2 (*n* = 67 cells). Similar results were obtained for the neutrophils (Supplementary Figure 1A) and the X^0^-CGD PLB-985 GFP-NOX2 cells (data not shown). We then examined the localization of NOX2 in Rab11-positive endosomes. Rab11 and its effectors define the endosomal recycling compartment. Rab11 is also involved in transport of cargo from the Golgi to the plasma membrane (Vale-Costa and Amorim, 2016). Rab11-positive structures were localized close to the plasma membrane in PLB-985 WT cells. NOX2 was detected in 21.7% (*n* = 63 cells) of Rab11-positive endosomes. The Pearson coefficient was 0.45 (±0.05) indicating partial co-localization (Figure 3A). Similar results were obtained for neutrophils (Supplementary Figures 1A, 2A) and X^0^-CGD PLB-985 GFP-NOX2 cells (data not shown). Thus, part of NOX2 is localized in a fraction of early and recycling endosomes.

### Some EEA1 and Rab11-Positive Endosomes Are Found Close to the Phagosome

To ascertain whether endosomes are able to deliver NOX2 to the phagosome, we examined the localization of endosomes after 10 min of opsonized zymosan phagocytosis in PLB-985 WT cells and in neutrophils. We observed both EEA1- and Rab11-positive endosomes close to the phagosomes (Figures 2B, 3B and Supplementary Figures 1B, 2B). Some of these endosomes were also positive for NOX2. Indeed, the fluorescence profiles of NOX2 and EEA1 or Rab11 around the phagosome show overlapping peaks, indicating co-localization, in some spots (Figures 2B, 3B and Supplementary Figures 1B, 2B). The same results were obtained for the X^0^-CGD PLB-985 GFP-NOX2 cells (data not shown). Our data suggest that a proportion of the total NOX2 is localized in certain EEA1- and Rab11-positive endosomes and that these endosomes may fuse with the phagosome to convey new NOX2 molecules to the phagosomal membrane.

### NOX2 Forms Clusters and Their Number Increases During Phagocytosis

**FIGURE 4:**
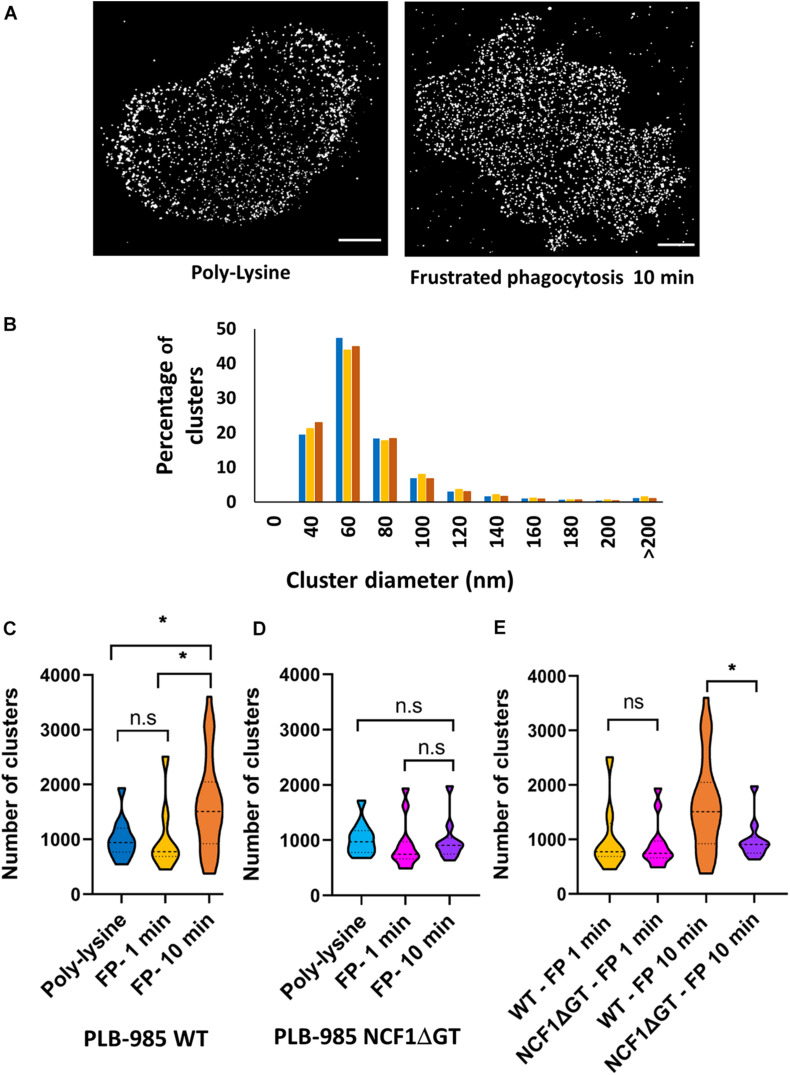
The number of NOX2 nanoclusters increases during frustrated phagocytosis in PLB-985 WT cells but not in PLB-985 NCF1ΔGT cells. Cells were incubated on poly-lysine or for 1 or 10 min on IgG-coated coverslips. The latter conditions promoted frustrated phagocytosis. Following fixation and permeabilization, NOX2 labeling was observed using dSTORM in a TIRFM configuration. **(A)** Representative images of NOX2 in PLB-985 WT cells coated on poly-lysine or following 10 min on IgG-coated coverslips. **(B)** Clusters of NOX2 were detected using SR-Tesseler software. The graph illustrates the distribution of NOX2 nanoclusters according to size on the PLB-985 WT cell surface in the different conditions: non-activated (poly-L-lysine, blue, 11 cells) or after 1 min (yellow, 8 cells) and 10 min (red, 28 cells) of frustrated phagocytosis. Three independent experiments. **(C,D)** Numbers of NOX2 nanoclusters on the cell surface in the different conditions described above for PLB-985 WT cells **(C)** and PLB-985 NCF1ΔGT cells **(D)**. For D, the conditions are the same as C: non-activated cells (cyan, 10 cells), 1 min (pink, 12 cells) or 10 min (purple, 10 cells) of frustrated phagocytosis, three independent experiments. FP: frustrated phagocytosis. **(E)** Comparison between the number of clusters in PLB-985 WT and PLB-985 NCF1ΔGT cells during frustrated phagocytosis for 1 or 10 min. Each violin plot represents the spread of the values. The interquartile range and medians are represented as dotted line. *p < 0.05; ns: non-significant (Mann Whitney tests).

The phagocytic cup has a spatial and temporal arrangement of receptors and signaling molecules (Goodridge et al., 2011; Freeman et al., 2016) that is similar to the immunologic synapse (Yokosuka et al., 2005). This specific arrangement of molecules at the phagocytic cup is known as “phagocytic synapse” (Niedergang et al., 2016). The spatial arrangement of NOX2 at the phagocytic cup is as yet unknown. To gain insight into NOX2 spatial organization and dynamics in the membrane during phagocytosis, we used a frustrated phagocytosis paradigm in which the cells are allowed to spread on IgG-coated coverslips (Marion et al., 2012). The PLB-985 WT cells were activated in this manner for 1 and 10 min. For the control condition (non-activated), poly-L-lysine-coated coverslips were used. The cells were then stained for NOX2 by immunofluorescence and imaged using a dSTORM approach with a TIRFM configuration. TIRFM allows the imaging of the fluorophores in the membrane in proximal contact with the coverslip while avoiding potentially confusing contribution from cytoplasmic fluorophores. A density-based representation of dSTORM images revealed that NOX2 distribution was similar in the 3 conditions: non-activated, frustrated phagocytosis for 1 or 10 min (Figure 4A and data not shown). The spatial distribution of NOX2 analyzed using the DBSCAN cluster detection analysis implemented in SR-Tesseler software (materials and methods) indicated that NOX2 organized in nanoclusters. These clusters had a similar size distribution in the cell membrane in each condition (Figure 4B). The mean diameter of the nanoclusters was about 60 nm in each condition (60.5 ± 7.1 nm in the non-activated condition; 66.2 ± 10.5 nm after 1 min of frustrated phagocytosis, 63.6 ± 17.25 nm after 10 min of frustrated phagocytosis). In each condition, almost all the fluorophores detected following the analyses of dSTORM images (materials and methods) were found inside clusters, and the number of fluorophores per cluster was similar (Supplementary Figure 3). However, a significant rise in the number of clusters was observed in the PLB-985 WT cells after 10 min of phagocytosis as compared to that after 1 min of phagocytosis or in non-activated cells (Figure 4C). This increase was correlated with a larger frustrated phagosomal surface after 10 min as compared to the surface observed in the 2 othet conditions (Supplementary Figure 5A). It has been shown that this spreading of the phagosomal surface during frustrated phagocytosis is not only due to a passive membrane extension, but that it also involved the delivery of new membranes (Zak, 2019, manuscript in preparation). Thus, this increase in the number of clusters indicated that new NOX2 molecules were delivered to the membrane between 1 and 10 min after the start of frustrated phagocytosis.

### The Increase in the Number of NOX2 Clusters During Phagocytosis Requires p47^phox^

In order to know whether the increase in NOX2 clusters requires a functional oxidase, we performed the same experiments as before using PLB-985 NCF1ΔGT cells, which lack a functional oxidase since they are deficient for p47^phox^ (Wrona et al., 2017). The distribution of NOX2 nanocluster sizes in these cells was the same as for the WT cells in the 3 conditions (Supplementary Figure 4), as was the number of fluorophores per clusters (data not shown). However, in contrast to the results obtained using WT cells, in the cells lacking a functional oxidase no difference was detected either in the number of clusters or in the frustrated phagosomal surface after 10 min of phagocytosis compared to that after 1 min of phagocytosis or in non-activated cells (Figure 4D and Supplementary Figure 5B). The number of clusters was significantly different, after 10 min of phagocytosis, between PLB-985 NCF1ΔGT cells and PLB-985 WT cells (Figure 4E). Thus, taken together, our data indicate that the presence of p47^phox^ and/or ROS production are required for the delivery of NOX2 clusters during frustrated phagocytosis.

## Discussion

In this study, we have examined for the first time the spatial arrangement of NOX2 in the phagosome membrane using super-resolution microscopy. This has revealed that NOX2 molecules are organized in nanoclusters. These clusters increased during frustrated phagocytosis, indicating the delivery of NOX2 to the phagosomal membrane. This delivery required the presence of p47^phox^.

We used X^0^-CGD PLB-985 GFP-NOX2 cells to follow the modifications of NOX2 in the phagosomal membrane during phagocytosis. We observed an accumulation of NOX2 compared to the plasma membrane just after phagosome closure. Using immunofluorescence, we detected that NOX2 was localized in a fraction of the EEA1- and Rab11-positive organelles in PLB-985 cells and also in neutrophils. It is of interest to note that no co-localization with the lysosome marker LAMP1 could be detected (data not shown). Some of these organelles were positioned close to the phagosomes after 10 min of phagocytosis, suggesting their involvement in the delivery of new NOX2 molecules to the phagosome in the PLB-985 cells as well as in neutrophils.

These results are also coherent with the super-resolution data in which we observed an increase in the frustrated phagosomal surface and in the number of NOX2 nanoclusters during phagocytosis. These nanoclusters have a random distribution in the membrane, unlike receptors such as Dectin, FcRs and the phosphatase CD45 at the beginning of phagocytosis (Goodridge et al., 2011; Freeman et al., 2016). This cluster organization of NOX2 was previously observed by Wientjes et al. (1997) using immuno-electronmicroscopy. These investigators observed nanodomains with a diameter of 200–360 nm. This size is larger than the one we detected (Figure 4B), although the discrepancy can be explained by the different techniques used. In the future, double labeling experiments should reveal, which proportion of these clusters contains cytosolic subunits and produces ROS.

A nanocluster organization has been observed for many membrane proteins. Garcia-Parajo et al. (2014) proposed several hypotheses to explain this organization, one being that it may facilitate ligand binding. In our case, it could facilitate the binding of the cytosolic subunits and Rac to the heterodimer NOX2-p22^phox^ when the neutrophils are activated. The average diameter of NOX2 clusters was 60 nm. Similar cluster size has been reporter for other proteins such as the z chain of CD3, which is the co-receptor of the T-cell receptor (Garcia-Parajo et al., 2014; Pageon et al., 2016). However, upon activation of the T-cell receptor, CD3 formed clusters of 185 nm in diameter (Pageon et al., 2016). In the case of NOX2, neither the cluster size nor the number of fluorophores per cluster (about 100) changed during phagocytosis. If we assume that we have around 5 fluorophores attached to each secondary antibody, and two secondary antibodies per primary antibody against NOX2, then we would have an average of 10 NOX2 molecules per cluster. A similar result has been found for the dendritic cell receptor DC-SIGN (Garcia-Parajo et al., 2014).

In PLB-985 WT cells an increase in the number of clusters occurred between 1 min and 10 min after the start of phagocytosis, indicating that new NOX2 molecules were delivered to the membrane. The increase may be explained by the fusion of endosomes containing NOX2 with the phagosomal membrane. The NOX2 clusters might already be formed in the endosomal membrane. We don’t know whether NOX2 is already assembled with the cytosolic subunits and active inside the endosomes. Such an endosomal ROS production has been described upon endotoxin priming in neutrophils (Lamb et al., 2012). Moreover, the specific granules have also described to be site of ROS production (Karlsson and Dahlgren, 2002). Anderson et al. observed ROS production in extra-phagosomal sites upon phagocytosis and in reponse to Fcg receptor stimulation (Anderson et al., 2010). It would be of interest to examine whether ROS production is detectable at endosomal sites and what could be the functions of this ROS production.

Neither the number of clusters nor the degree of cell spreading were modified between 1 and 10 min of phagocytosis in the PLB-985 NCF1ΔGT cells lacking p47^phox^. Thus, p47^phox^ may have a structural role in the recruitment of NOX2 to the phagosome. Alternatively, as these cells lack a functional NADPH oxidase, ROS may be necessary for the delivery of new NOX2 clusters at the phagosome. In order to check this hypothesis, PLB-985 cells expressing inactive mutants of NOX2 (Picciocchi et al., 2011) would be appropriate.

Thus, NADPH oxidase activity may trigger a positive feedback with the recruitment of additional NOX2 positive vesicles. One hypothesis to explain this phenomenon would be that ROS allow the sustained activation of kinases like Erk (Ray et al., 2012) by inactivating protein tyrosine phosphatases such as PTP1B. The Erk kinase favors exocytosis from the Rab11-recycling compartment (Robertson et al., 2006). Thus, a sustained activation of Erk would allow the fusion of Rab-11 vesicles with the phagosome. Further work will be necessary to investigate this hypothesis i.e., the involvement of ROS in membrane fusion in PLB_985 cells but also in neutrophils, which have specific granules containing NOX2.

## Data Availability Statement

The original contributions presented in the study are included in the article/Supplementary Material, further inquiries can be directed to the corresponding author/s.

## Author Contributions

JJ, SD-C, and ON designed the research. SD-C supervised the research project. JJ and EH performed the experiments. JJ set up the experiments with the help of SL for the dSTORM and JJ analyzed the experiments. PV and DR constructed the X^0^-CGD PLB-985 GFP-NOX2 cells. DW, US, and JR constructed the PLB-985 NCF1ΔGT cells. SD-C wrote the manuscript with the help of JJ. ON and DR commented on the manuscript. All authors contributed to the article and approved the submitted version.

## Conflict of Interest

The authors declare that the research was conducted in the absence of any commercial or financial relationships that could be construed as a potential conflict of interest.

## Abbreviations

dSTORM: direct stochastic optical resolution microscopy
DBSCAN: density based spatial clustering of applications with noise
EEA1: early endosome antigen1
IgG: Immunoglobulin G
PC: Pearson coefficient
ROS: reactive oxygen species
TIRFM: total internal reflection fluorescence microscopy.
