# Supplementary material for: Membrane Dynamics and Organization of the Phagocyte NADPH Oxidase in PLB-985 Cells
Source: Front Cell Dev Biol. 2020 Nov 12;8:608600. doi: 10.3389/fcell.2020.608600 (PMC7751761; doi:10.3389/fcell.2020.608600)
Supplement: Supplementary file 1 [file Presentation_1.pdf]

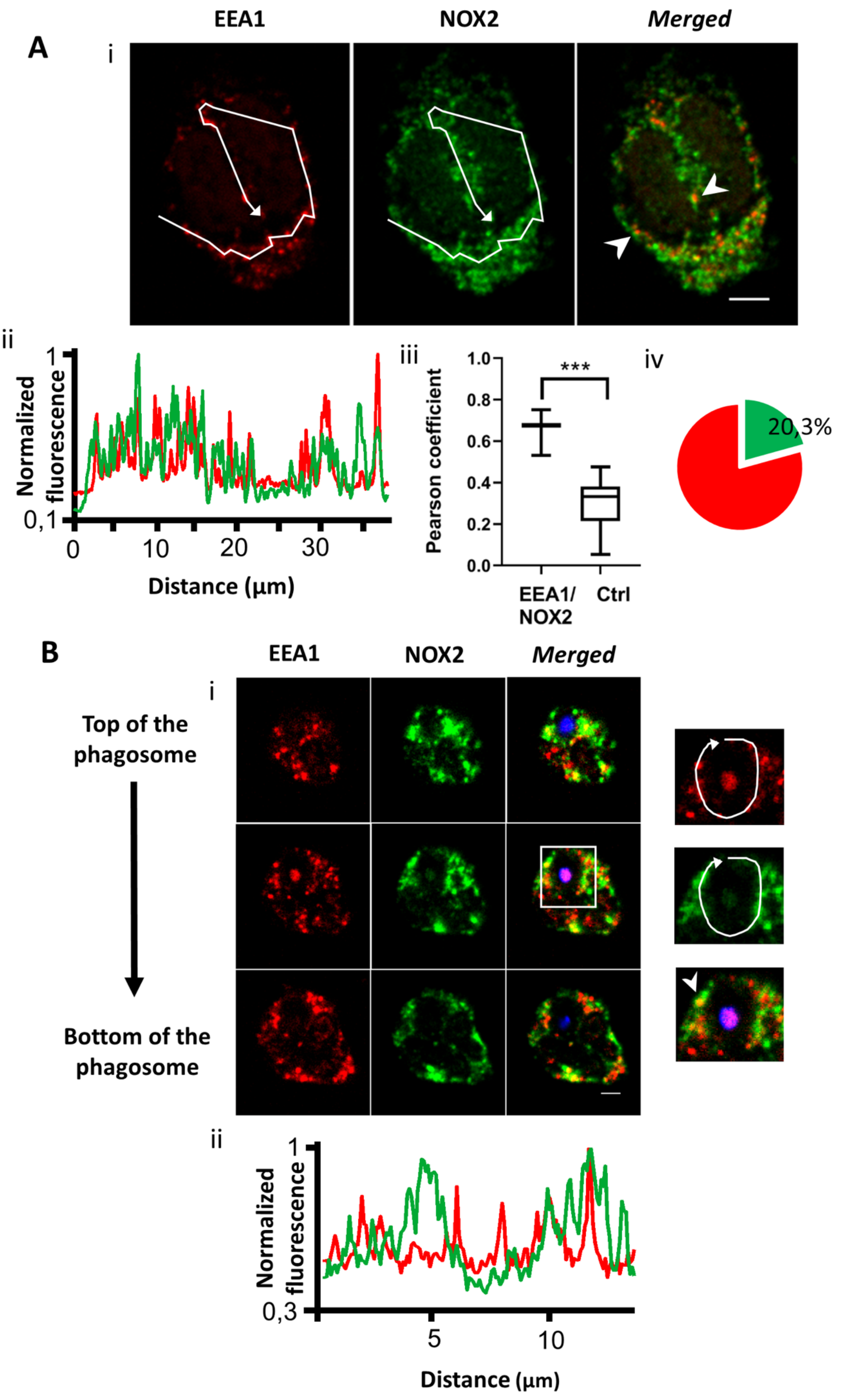

Supplemental Figure 1

Supplemental figure 1: **NOX2 is present in some EEA1-positive endosomes that are localized close to the phagosome during phagocytosis in neutrophils.**

(A) EEA1 (red) and NOX2 (green) were detected by immunofluorescence in resting neutrophils using spinning disk confocal microscopy. (Ai) Representative images of single planes from a Z-stack. Scale bar = 3 µm. Three independent experiments. (Aii) Normalized fluorescence of EEA1 and NOX2 along the white arrow shown in Ai. Overlapping peaks of fluorescence indicate co-localization of NOX2 and EEA1. (Aiii) Co-localization between EEA1 and NOX2 estimated using the Pearson coefficient; PCs correlating EEA1 and NOX2 images (EEA1/NOX2) were controlled by comparison with the PCs correlating the EEA1 image and the image of NOX2 rotated by 180° (Control, Ctrl). Twelve cells from three independent experiments were analyzed. Each boxplot represents inter-quartile range with the median, *** represents $p<0.005$ (Mann Whitney test). (Aiv) Percentage of EEA1-positive endosomes containing NOX2 determined with "object-based method" using Image J plug-in JACoP.

(B) EEA1 (red) and NOX2 (green) were detected by immunofluorescence after 10 min of phagocytosis with opsonized Texas Red-zymosan. (Bi) Three planes from a Z-stack series (0.5 µm). Some EEA1 dots were observed close or at the phagosomes. Scale bar = 3 µm. Two independent experiments. (Bii) Normalized fluorescence of EEA1 and NOX2 along the white arrow showing some overlapping peaks of NOX2 and EEA1 fluorescence at the phagosome.

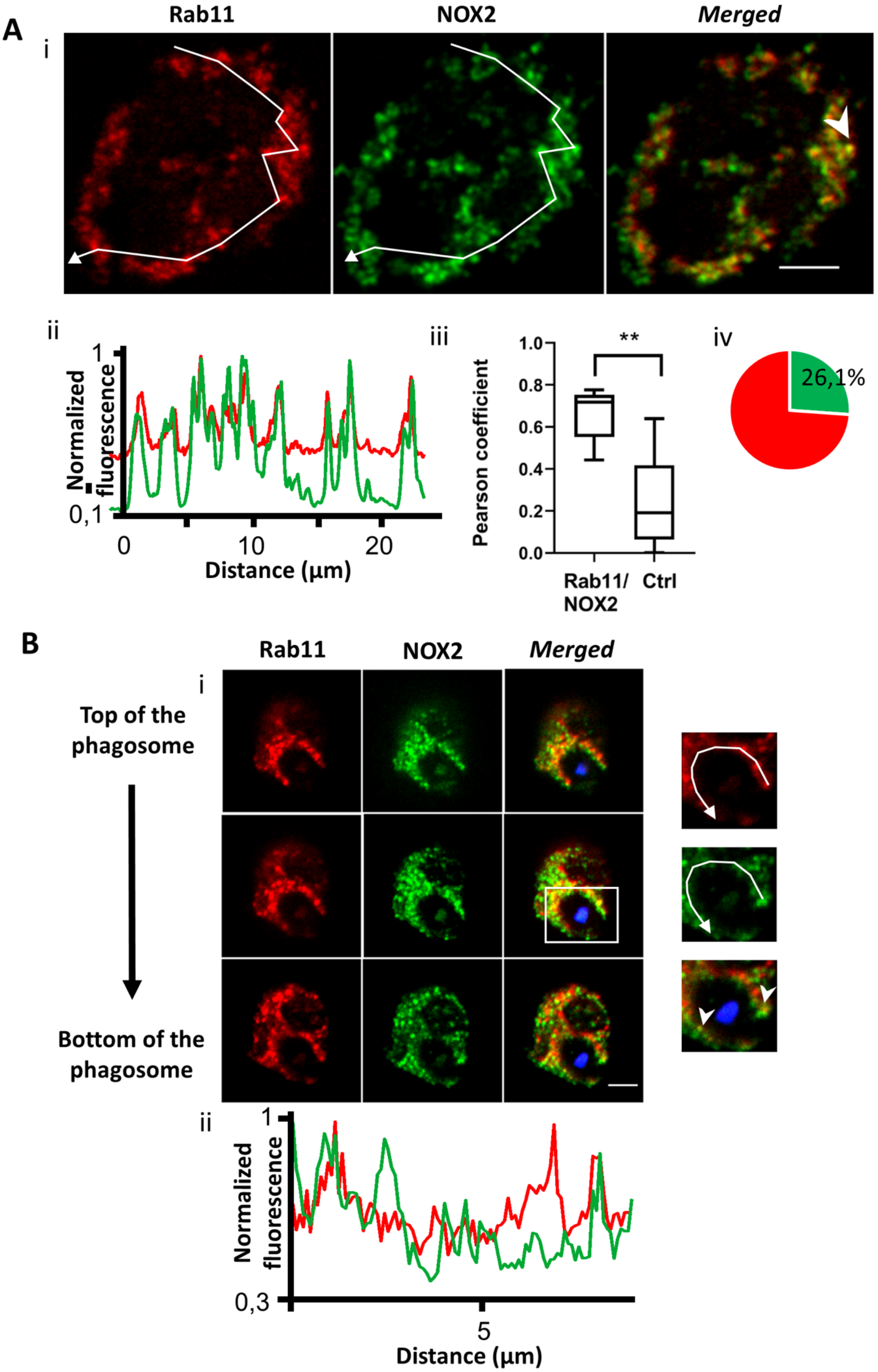

Supplemental Figure 2

Supplemental figure 2: **NOX2 is present in some Rab11-positive endosomes that are localized close to the phagosome during phagocytosis in neutrophils.**

(A) Rab11 (red) and NOX2 (green) were detected by immunofluorescence in resting neutrophils using spinning disk confocal microscopy. (Ai) Representative images of single planes from a Z-stack. Scale bar = 3 µm. Three independent experiments. (Aii) Normalized fluorescence of Rab11 and NOX1 along the white arrow shown in Ai. Overlapping peaks of fluorescence indicate co-localization of NOX2 and Rab11. (Aiii) Co-localization of Rab11 and NOX2 estimated using the Pearson coefficient (PC). PCs correlating Rab11 and NOX2 images (Rab11/NOX2) were controlled by comparison with the PCs correlating the Rab11 image and the image of NOX2 rotated by 180° (Control, Ctrl). Nine cells from three independent experiments were analyzed. Each boxplot represents inter-quartile range with the median, ** represents $p<0.01$ (Mann Whitney test). (Aiv) Percentage of Rab11-positive endosomes containing NOX2 determined with “object-based method” using Image J plug-in JACoP. (B) Rab11(red) and NOX2 (green) were detected by immunofluorescence after 10 min of phagocytosis with opsonized Texas Red-zymosan. (Bi) Three planes from a series of Z-stack planes (0.5 µm). Some Rab11-positive structures were observed close to or at the phagosomes. Scale bar = 3 µm. Two independent experiments. (Bii) Normalized fluorescence of Rab11 and NOX2 along the white arrow showing some overlapping peaks of NOX2 and Rab11 fluorescence at the phagosome.

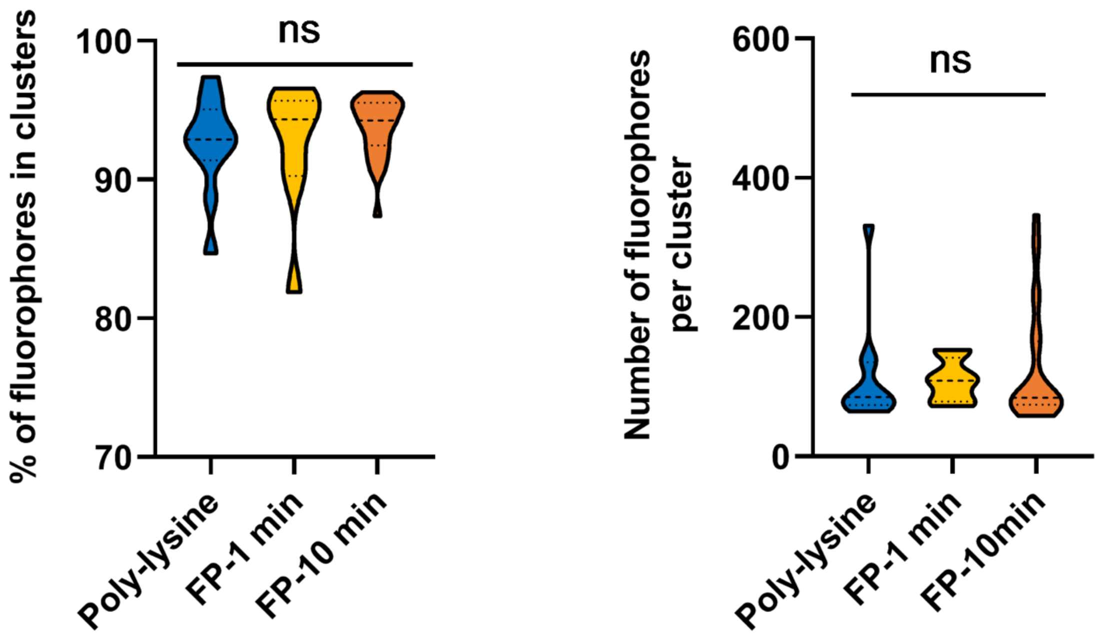

Supplemental figure 3: **Clustering parameters extracted from DBSCAN analysis in PLB-985 WT cells** (A) Percentage of fluorophores detected inside clusters in the different conditions: cells coated on poly-L-lysine (blue, 11 cells), during frustrated phagocytosis after 1 min (yellow, 8 cells) and after 10 min (orange, 28 cells). (B) Number of fluorophores per cluster in the above conditions. Three independent experiments. Each violin plot represents the spread of the values. ns: non-significant. (Kruskal Wallis test)

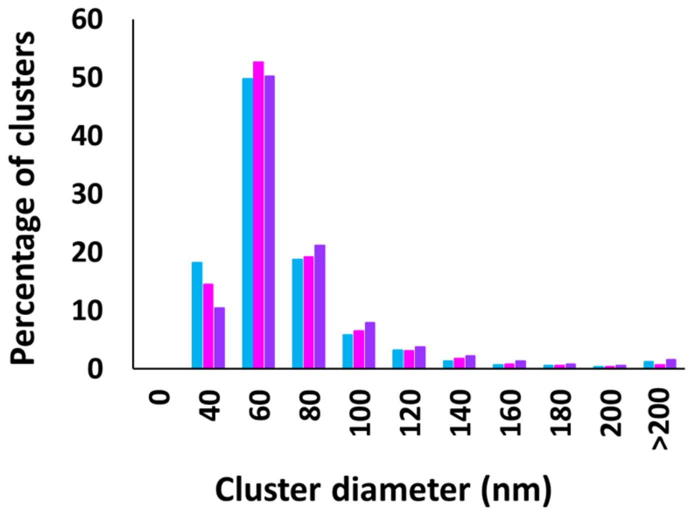

Supplemental figure 4: **NOX2 nanocluster sizes on the PLB-985 NCF1ΔGT cell surface**
The cells have been incubated on poly-L-lysine or on IgG-coated coverslips for 1 or 10 min. The latter conditions allowed frustrated phagocytosis. Cells were then fixed, permeabilized and stained for NOX2. NOX2 was observed using dSTORM in TIRFM configuration. Distribution of NOX2 clusters on PLB-985 NCF1ΔGT cell surface, according to their size in the different conditions: cells coated on poly-L-lysine (blue, 10 cells), frustrated phagocytosis for 1 min (pink, 12 cells) and for 10 min (purple, 10 cells). Three independent experiments.

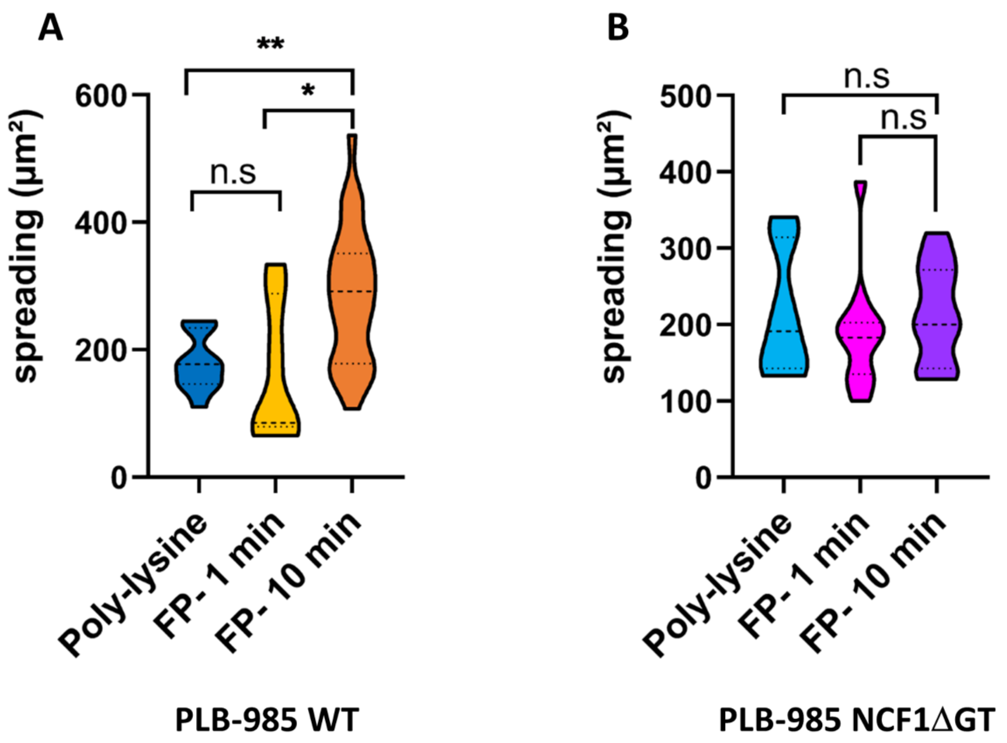

Supplemental figure 5: **Frustrated phagosomal surface increases during frustrated phagocytosis in PLB-985 WT cells but not in PLB-985 NCF1ΔGT cells.**

The cells were incubated either on poly-L-lysine or on IgG-coated coverslips for 1 or 10 min. The latter conditions allowed frustrated phagocytosis. Cells were then fixed, permeabilized and stained for NOX2. NOX2 was observed using dSTORM in TIRFM configuration. (A, B) Frustrated phagosomal surface or cell surface spreading (μm^2) in the different conditions described above in PLB-985 WT cells (A) and PLB-985 NCF1ΔGT cells (B). Each violin plot represents the spread of the values and the medians. * $p<0.05$, ** $p<0.01$, ns: non-significant (Mann Whitney tests).
